# MRI-based assessment of the pineal gland in a large population of children aged 0–5 years and comparison with pineoblastoma: part I, the solid gland

**DOI:** 10.1007/s00234-016-1684-z

**Published:** 2016-04-29

**Authors:** Paolo Galluzzi, Marcus C. de Jong, Selma Sirin, Philippe Maeder, Pietro Piu, Alfonso Cerase, Lucia Monti, Hervé J. Brisse, Jonas A. Castelijns, Pim de Graaf, Sophia L. Goericke, On behalf of the European Retinoblastoma Imaging Collaboration (ERIC)

**Affiliations:** Unit of Diagnostic and Therapeutic Neuroradiology, Department of Neurosciences, Azienda Ospedaliera Universitaria Senese, Siena, Italy; Department of Radiology and Nuclear Medicine, VU University Medical Center, PO Box 7057, 1007 MB Amsterdam, The Netherlands; Department of Diagnostic and Interventional Radiology and Neuroradiology, University Hospital Essen, Essen, Germany; Department of Radiology, University Hospital, Lausanne, Switzerland; Department of Medicine, Surgery, and Neuroscience, University of Siena, Siena, Italy; Department of Radiology, Institut Curie, Paris, France

**Keywords:** Pineal gland, Pineoblastoma, Retinoblastoma, Pediatric, Gland size

## Abstract

**Introduction:**

Differentiation between normal solid (non-cystic) pineal glands and pineal pathologies on brain MRI is difficult. The aim of this study was to assess the size of the solid pineal gland in children (0–5 years) and compare the findings with published pineoblastoma cases.

**Methods:**

We retrospectively analyzed the size (width, height, planimetric area) of solid pineal glands in 184 non-retinoblastoma patients (73 female, 111 male) aged 0–5 years on MRI. The effect of age and gender on gland size was evaluated. Linear regression analysis was performed to analyze the relation between size and age. Ninety-nine percent prediction intervals around the mean were added to construct a normal size range per age, with the upper bound of the predictive interval as the parameter of interest as a cutoff for normalcy.

**Results:**

There was no significant interaction of gender and age for all the three pineal gland parameters (width, height, and area). Linear regression analysis gave 99 % upper prediction bounds of 7.9, 4.8, and 25.4 mm^2^, respectively, for width, height, and area. The slopes (size increase per month) of each parameter were 0.046, 0.023, and 0.202, respectively. Ninety-three percent (95 % CI 66–100 %) of asymptomatic solid pineoblastomas were larger in size than the 99 % upper bound.

**Conclusion:**

This study establishes norms for solid pineal gland size in non-retinoblastoma children aged 0–5 years. Knowledge of the size of the normal pineal gland is helpful for detection of pineal gland abnormalities, particularly pineoblastoma.

**Electronic supplementary material:**

The online version of this article (doi:10.1007/s00234-016-1684-z) contains supplementary material, which is available to authorized users.

## Introduction

Three to four percent of patients with hereditary retinoblastoma also develop pineoblastoma (trilateral retinoblastoma) [1]. Before the age of 5 years, 95 % of all pineoblastomas are diagnosed [2]. Unfortunately, most patients with pineoblastoma die from this disease [2]. Asymptomatic pineoblastoma patients have shown a much better survival compared to symptomatic pineoblastoma, and the same was seen in small (≤15 mm) versus large (>15 mm) tumors [2]. Asymptomatic patients showed a 5-year survival of 50 %, whereas in patients with symptomatic disease, only 4 % survived [2], emphasizing the importance of early detection. Guidelines advise to perform magnetic resonance imaging in all newly referred retinoblastoma patients and to also include a scan of the entire brain [3].

Unfortunately, however, it is quite challenging to differentiate between a normal pineal gland and pineoblastoma, especially since the best prognosis after pineoblastoma is for patients with small tumors [4–6]. This leaves a small size window in which pineoblastoma should be diagnosed, therefore knowledge about the normal size range at the different ages of the pineal gland is important.

Besides pineoblastomas, knowledge of the normal pineal gland size in children is helpful for detecting abnormal glands and other pineal neoplasms [7]. Actually, small pineal tumors are often difficult to distinguish from normal tissue, due to a similar signal intensity compared to the gland itself and the physiologic enhancement after contrast medium injection due to the lack of a blood brain barrier. On MRI, the gland is usually isointense compared to grey matter on both T1- and T2-weighted images [8, 9]. Pineal gland growth in the first years of life has been documented [10, 11]. Cysts in the pineal gland are a common finding (25–41 % of otherwise normal glands of adolescent and adults at autopsy) [11, 12]; they occur at all ages, from the fetal period to senility [11, 13–18]. Few studies published in literature evaluated the range of the pineal size at different ages. In necropsy-based studies, some authors found a correlation between age and increase of pineal weight, sometimes related to sex, body weight, and different decades [14, 19, 20]; others did not find any correlation [21]. Most of the hitherto performed MRI studies used an inadequately high slice thickness that did not allow for a reliable differentiation of parenchymal and cystic components [10]. Moreover, MRI studies have characterized the pineal gland mainly in adults whereas only sparse information is available for the pediatric pineal gland.

We hypothesize that abnormal growth of the pineal parenchyma has to be considered a far more alerting sign in comparison to presence of cysts or their increase in size. Moreover, correlation of pineal size with age in children is stronger if only the solid part of the gland is measured [10]. Regarding the normal range of the pineal size, only sparse data exist in the literature concerning the in vivo microstructure and volume of the pineal gland in adults and even less is known in children [10, 12]. A recent study showed that there is no difference in pineal gland size of retinoblastoma versus non-retinoblastoma patients allowing for usage of the results from this paper in retinoblastoma patients as well [22].

The first goal of our study was to determine the normal growth pattern of solid pineal glands in a large population, aged 0–5 years. The second goal was to compare pineoblastoma cases from the literature with normal pineal gland sizes. The cystic pineal gland will be analyzed in part II of this study.

## Materials and methods

This retrospective study included patients from four European neuroimaging or radiological university centers in Amsterdam, Essen, Lausanne, and Siena. After excluding patients with any known endocrinologic or neurologic disorders (possibly) affecting or related to the pineal gland and distortion of pineal region from adjacent pathologies or artifacts hampering the evaluation of the gland, as well as those patients who were undergoing radiation therapy or chemotherapy, we retrospectively reviewed 184 (Siena *n* = 39, Essen *n* = 121, Lausanne *n* = 17, and Amsterdam *n* = 7) consecutive non-retinoblastoma patients (73 female, 111 male) in the age range from 0 to 5 years who underwent MR imaging from July 2005 through January 2015 in whom a solid (non-cystic) pineal gland could be clearly identified. In these patients, MRI was performed because of conditions that were not related to the pineal gland; the children were mainly affected by seizures, hydrocephalus, prematurity, development retardation, brain malformations, and neonatal asphyxia.

### Imaging

Due to the multicenter setting of this study, the examinations were performed on different 1.5 and 3.0 Tesla MR systems (Magnetom Avanto, Aera, Symphony or Skyra, Siemens Healthcare, Erlangen, Germany) and different T2-weighted sequences were used. We only included MR examinations if the sagittal T2-weighted sequences had a slice thickness of no more than 2 mm to minimize partial volume effects. The slice thickness of the included patients varied between 0.6 and 2 mm.

### MR data analysis

**Fig. 1:**
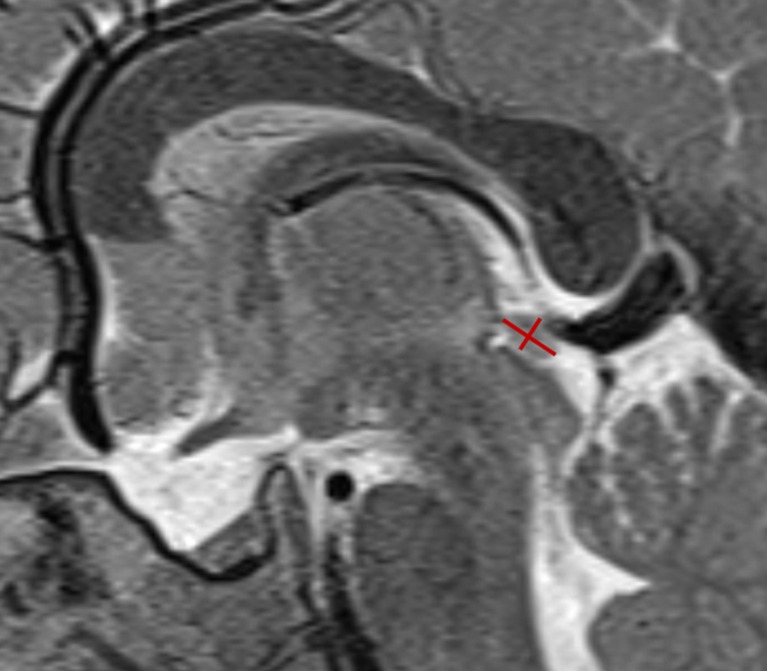
The largest antero-posterior (width) and cranio-caudal (height) diameters of the pineal gland were measured as shown in this sagittal T2-weighted image

The datasets were anonymized prior to analysis. Pineal size was estimated measuring its largest antero-posterior (width) and supero-inferior (height) diameters on the sagittal T2-weighted sequences (as shown in Fig. 1) and by calculating their planimetric area according to the formula: (width / 2) ⨯ (height / 2) ⨯ π. Measurements were performed by four senior neuroradiologists (S.G., P.d.G., P.G., and P.M.) with 12, 12, 17, and 26 years of experience, respectively.

### Statistics

We calculated the interobserver agreement (intraclass correlation coefficient [ICC]) for the gland size parameters that were (blinded for the initial measurements) also measured by A.C., M.C.J., and S.S. on a random subset of the included patients (30 patients with a solid gland and 30 patients with a cystic gland). The Kolmogorov-Smirnov test was used to verify the null hypothesis that the variable age of female and male subsamples came from the same continuous distribution. Patients were assigned to one of three 20 months age categories (unlike in part II of this article, we chose not to assign patients to categories of 1 year because there was a very uneven distribution of patients across the age interval: 0–5 years).

The *χ*^2^ independence test was calculated comparing the frequencies of the three categories of age [0, 20), [20, 40), and [40, 60] months in female and male subsamples. Lavene’s test was used to verify the null hypothesis of equal variance (i.e., homoscedasticity) of the pineal measurements across the age intervals by gender. The variable area (square root) and width (natural log) were transformed in order to meet the homoscedasticity assumption. The pineal variables were then subjected to a two-way analysis of variance (ANOVA) test with gender (two levels) and the three age categories [0, 20), [20, 40), and [40, 60] months. Tukey’s honestly significant difference test was performed for the post hoc analysis. Simple linear regression analysis was performed to predict each pineal variable (area, width, and height) based on age.

In addition, 99 % predictive intervals around the mean were used to represent the range where single new observations of pineal parameters would likely fall given specified values of the variable age. Prediction intervals account for the variability around the mean response inherent in any prediction, so they addressed the issue of finding predicted outcomes of area, width, or height based on age.

All statistical calculations were performed using the Statistical Package for the Social Sciences Software Package (SPSS Statistics, version 20; IBM, Armonk, NY, USA) and R (version 3.2.2, 2015, The R Foundation for Statistical Computing).

### Comparison with pineoblastoma

To evaluate the clinical usefulness of these age-dependent prediction intervals, we combined the regression line of width (which is usually also the maximum diameter of the gland) with the maximum diameter of pineoblastoma from the meta-analysis by De Jong and colleagues [2] to single graphs. We differentiated between symptomatic and asymptomatic pineoblastoma, and between tumor type: solid, cystic, partially cystic, and unknown; the original articles of eligible pineoblastoma cases included in the meta-analysis were re-evaluated for this. Of most interest in this part will be the asymptomatic solid pineoblastomas, as they resemble the solid pineal glands closest in terms of size and appearance. In part II, a similar comparison will be made.

## Results

**Fig. 2:**
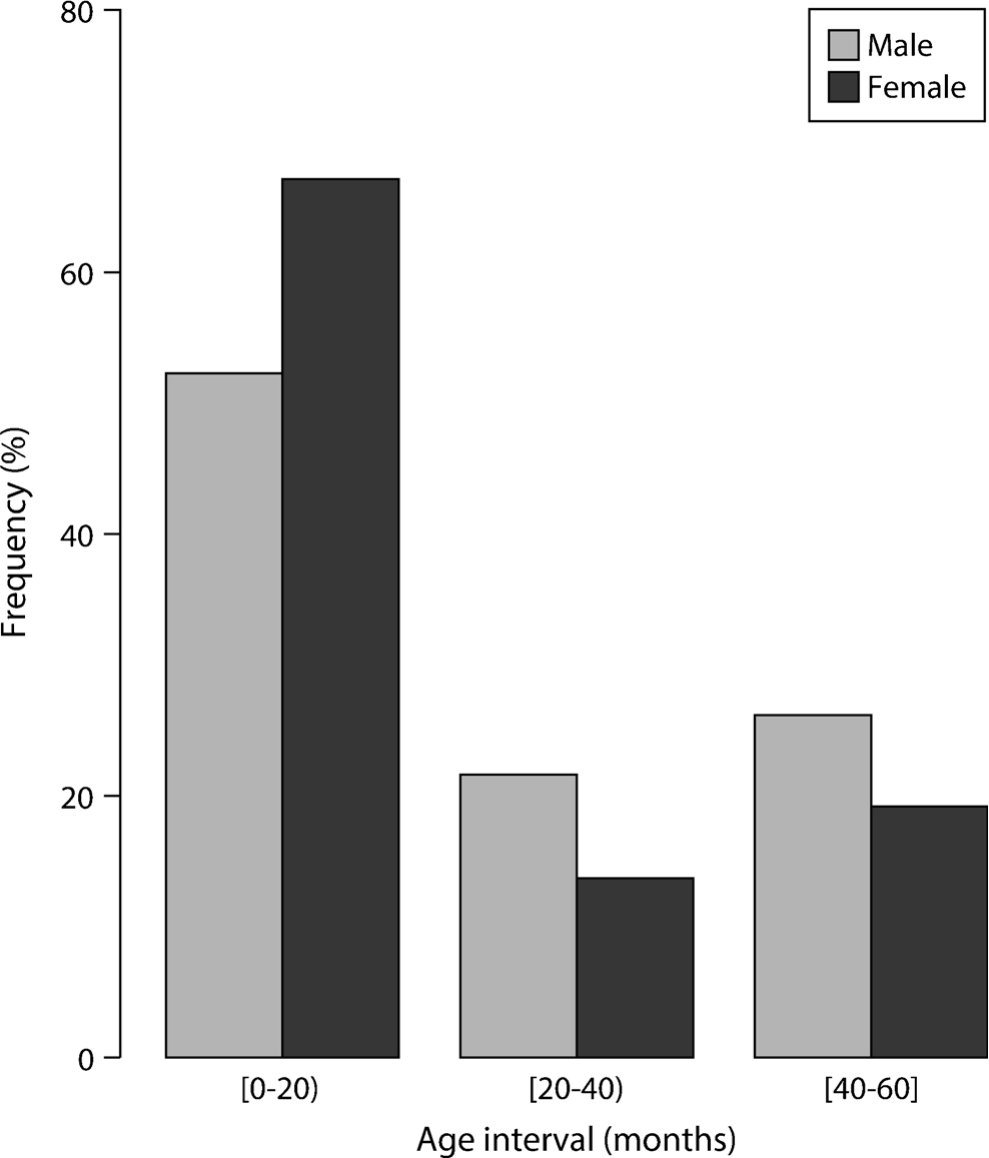
Frequency distribution of age by gender

Both gender-based subsamples showed an asymmetric distribution of age such that the age range [0, 20) months counted up to 67.1 and 52.2 % in female and male, respectively (Fig. 2).

The Kolmogorov-Smirnov test did not reject the null hypothesis that the variable age of female and male subsamples came from the same continuous distribution (*p* = 0.12). The chi-square test showed no significant interaction between age and gender, hence the age distribution did not relate significantly to gender (*p* = 0.083).

The width and height measurements showed ICCs of 0.996 (95 % confidence interval [CI] 0.991–0.998) and 0.996 (95 % CI 0.992–0.999), respectively.

### Analysis of variance

Lavene’s test showed *p* values of 0.21, 0.57, and 0.072 respectively for width, height, and area, implying that the group variances were not statistically heterogeneous and the usual ANOVA can be used. The results of ANOVA tests are displayed in Appendix A. The results of the ANOVA tests showed that age significantly predicted the three variables (width, height, and area). Neither gender nor the interaction effect of gender*age were statistically significant. In addition, the post hoc Tukey’s test suggested the presence of a shift in the evolution of the three parameters after the age of 20 months. Specifically, all the three pineal parameters evaluated in the first age interval [0, 20) were significantly lower than in the other age intervals (Appendix B).

### Linear regression analysis

**Fig. 3:**
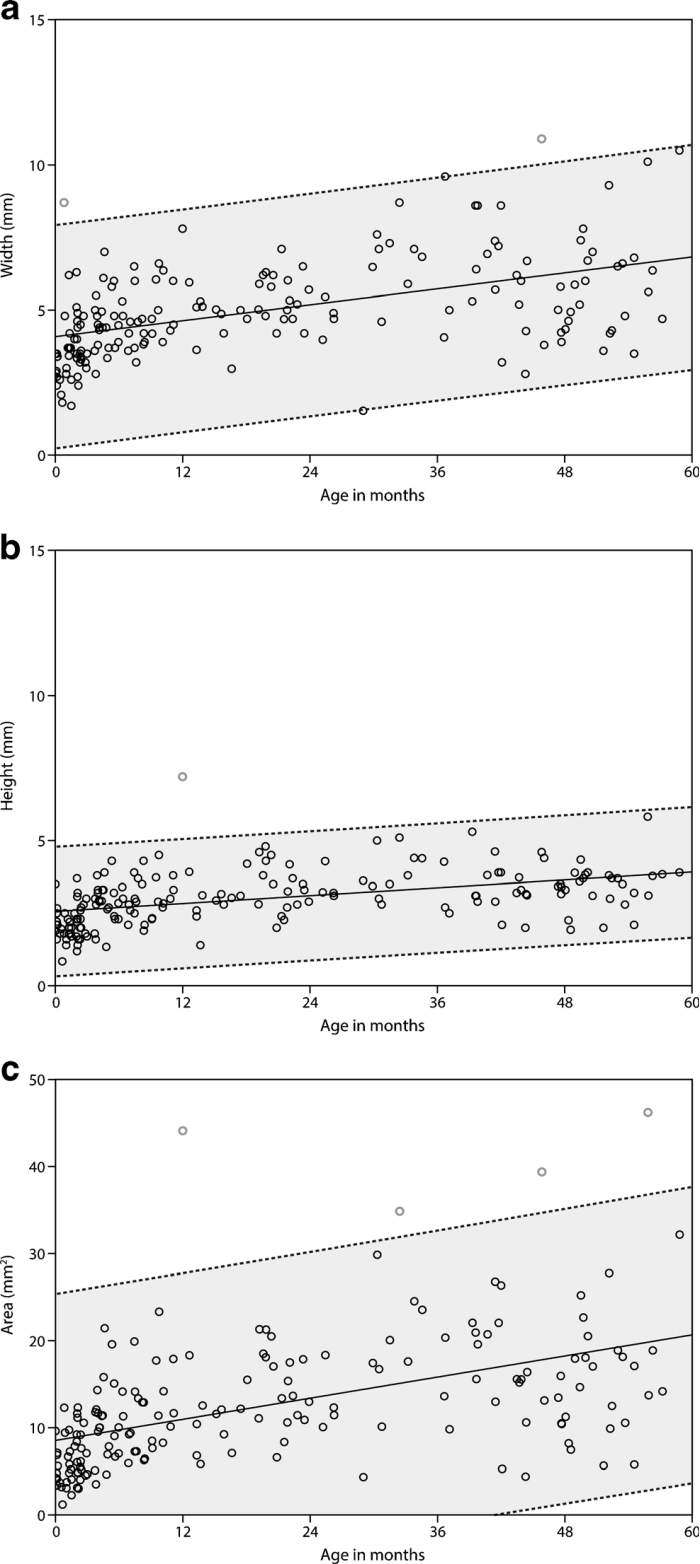
Linear regression of (**a**) width (mm), (**b**) height (mm), and (**c**) area (mm^2^) with 99 % prediction intervals from 0 to 60 months of age. The out-of-bound points are colored *red*

Linear regression of area on age significantly predicted area measurements (*p* < 0.0001; Table 1). Age also explained a significant proportion of the variance in area (adjusted *R*^2^ = 0.26; *F* test: *p* < 0.0001). Area (mm^2^) can be expressed by the equation 8.5 + 0.202 ⨯ age. This finding indicates that the size of area increased by 0.202 mm^2^ for each month of age starting with a mean area size of 8.5 mm^2^ at an age of 0 months. Of interest for the normal range of pineal gland size are the upper bounds of the 99 % prediction intervals. Thanks to the relatively large sample size, these 99 % prediction intervals approach linearity and a similar formula can be constructed. For area, the equation of the upper bound is then 25.4 + 0.202 ⨯ age. Similarly, significant regression equations were obtained for the other two relationships width versus age and height versus age, with an adjusted *R*^2^ of 0.25 (*F* test: *p* < 0.0001) and 0.20 (*F* test: *p* < 0.0001); width and height respectively gave the following formulas: 4.1 + 0.046 ⨯ age and 2.6 + 0.023 ⨯ age (see Table 1 and Fig. 3). For the 99 % upper bounds, 7.9 + 0.046 ⨯ age and 4.8 + 0.023 ⨯ age can be used as the formula respectively for width and height.

**Table 1:** Results of linear regression analysis: solid pineal gland size versus age

Relationship	Mean intercept (mm)	Upper bound (mm)^a^	Slope (mm/month)	*p* value	Adjusted *R* ^2^
Width vs. age	4.09	7.93	0.046	<0.0001	0.25
Height vs. age	2.56	4.80	0.023	<0.0001	0.20
Relationship	Mean intercept (mm^2^)	Upper bound (mm^2^)	Slope (mm^2^/month)	*p* value	Adjusted *R* ^2^
Area vs. age	8.53	25.41	0.202	<0.0001	0.26

^a^The upper 99 % prediction bound approaches linearity, and therefore, the slope of the linear regression line can be used

In multivariable linear regression analysis of all size parameters, gender was statistically insignificant (*p* values of 0.96, 0.11, and 0.30 respectively for width, height, and area) and was therefore not considered in further analysis. This result backed up that the progression of the pineal parameters with respect to age was independent of the gender of the patients.

Two out-of-bound points arose in the width versus age regression (Fig. 3a), resulting in a false-positive rate of 1.1 % (2/184). One out-of-bound point was found for the regression of height on age (false-positive rate 0.5 % HSC63227[1/184]; Fig. 3b). One point in the area versus age regression (Fig. 3c) lay beyond the upper bound, which yielded a false-positive rate of 2.2 % (4/184).

### Comparison with pineoblastoma

**Fig. 4:**
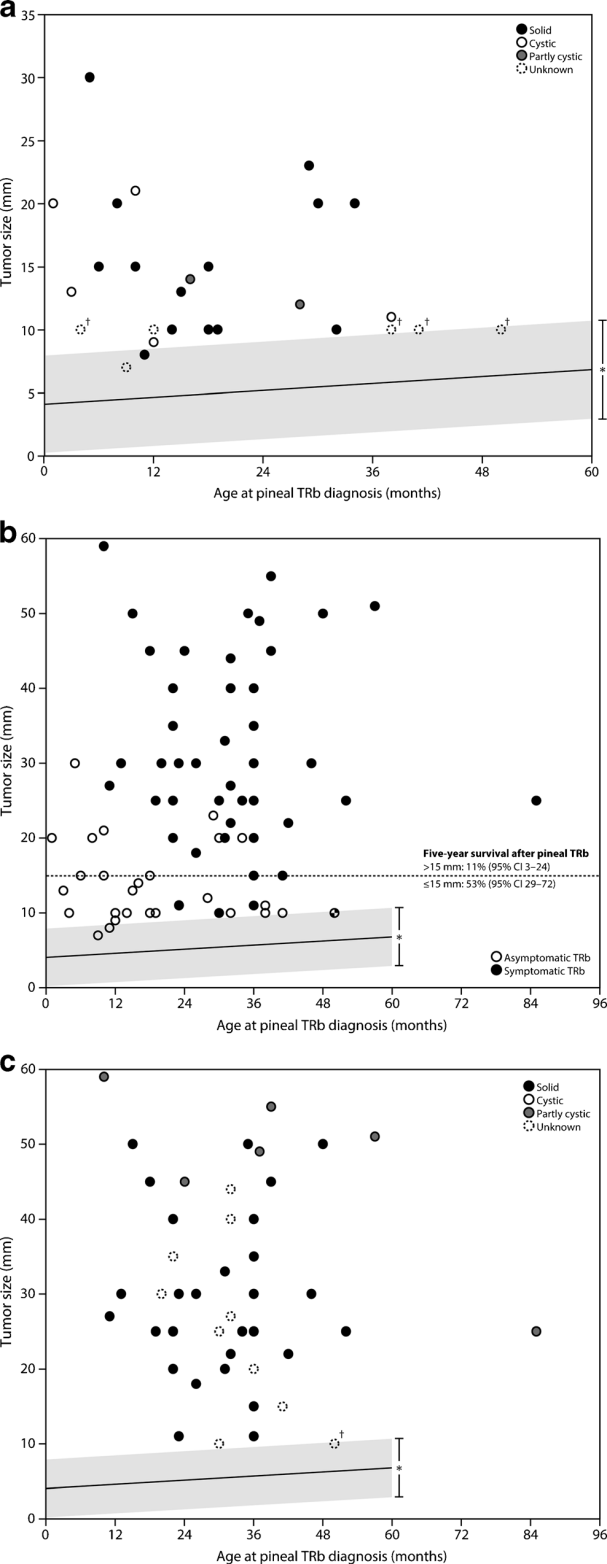
Regression line of the normal solid pineal gland width (mm) versus **a** maximum diameter (mm) of only asymptomatic pineoblastomas, **b** maximum diameter (mm) of both symptomatic and asymptomatic pineoblastomas, and **c** maximum diameter (mm) of only symptomatic pineoblastomas. *99 % prediction intervals. †These pineoblastomas are shown in the graph with a size of 10 mm, but had a reported size of 5–15 mm

In Fig. 4a, we plotted the linear regression line with 99 % prediction interval of the width (which is similar to the maximum diameter of the gland) of normal solid pineal glands (Table 1) together with the maximum diameter at time of diagnosis of the asymptomatic trilateral retinoblastomas as published in the meta-analysis by De Jong et al. [2] (one circle represents one pineoblastoma case, *n* = 27; see Fig. 4a). Eighty-nine percent (24 of 27; 95 % CI 71–89 %) of the pineal trilateral retinoblastoma cases, especially of interest, 93 % (13 of 14; 95 % CI 66–100 %) of the solid pineoblastomas, lie beyond the upper bound of normal pineal glands. Most pineoblastomas will not be symptomatic before they reach a certain size; see Fig. 4b where we plotted both the symptomatic (*n* = 44) and asymptomatic pineoblastomas (*n* = 27). Figure 4c shows which symptomatic pineoblastomas were solid or (partially) cystic.

## Discussion

The objective of this study was to provide normative values for solid pineal gland size by gender and age in non-retinoblastoma children from 0 to 5 years and to compare with pineoblastoma tumor size. Linear modeling was used to describe the relationship between solid pineal gland size and age. Age significantly explained the increase in the pineal gland size parameters width, height, and area independent of gender. Even though there were substantially less data points beyond the age of 20 months, the observed sample does support the hypothesis of a linear relationship between age and size (area, width, and height). Presentation of prediction intervals around the estimated mean values is informative about the ranges of the expected normal growth in the pineal gland size. Accordingly, new observations outside the bounds should be considered a warning signal of abnormality. Under this perspective, our study provides clinically useful backing material for interpretation of pineal gland growth in young children. A joint comparison of pineal size to the three prediction intervals of area, width, and height is recommended in order to get rid of ambiguous interpretation of the warning signals and for reducing the false-positive rate.

The pineal gland develops from an embryonic evagination of the third ventricle. In autopsy-based studies, available only in adults, reference values have been reported for pineal weight as well as approximated volumes [19, 21, 23]. Halfway the first decade of life, the structure of the pineal gland approaches that of a mature gland. If the cavum pineale is completely obliterated, the gland is “solid.” If the obliteration is incomplete, one or more cystic cavities remain, which are lined by cells that may differentiate to glial or ependymal cells. Parts of the cysts retain a connection with the ventricular system, which may induce their further enlargement [10, 13].

In 1995, Schmidt et al. were the first to estimate the pineal size by MRI in a large population of children aged 1 day to 15 years [12]. The authors found a mean transaxial diameter of 5.6 mm (SD 2.1), a midsagittal diameter of 5.0 mm (SD 2.4), and a planimetric area of 28.5 mm^2^ (SD 17.8) that did not change with age, and thus suggested a growth arrest of the pineal gland after infancy.

Sumida et al. [24] retrospectively studied by MRI a large population of patients aged 2 weeks to 20 years. In patients younger than 2 years, the mean size of the pineal gland was as follows: maximum length = 4.8 mm (SD 0.9) and height = 2.9 mm (SD 0.6). In patients aged 2 to 20 years, the gland size was larger and remained stable (average length = 6.1 mm [SD 1.2], average height = 3.7 mm [SD 0.8]). Compared to our study, they examined a smaller sample size of 63 patients in the age range of 0–5 years. The results of Schmidt et al. [12] and Sumida et al. [24] could overestimate the size of the glands because of the inclusion of cystic glands in their series; actually, asymptomatic pineal cyst may exert an important influence on pineal size. A serial MRI study performed by Barboriak et al. [16] in a small series over a period of 6 months to 9 years confirmed that pineal cysts remain unchanged on the whole and that cysts can either form or involute in individual patients. For more information about the cystic pineal gland, please see part II.

Recently, Bumb et al. [10] evaluated the correlation between pineal gland volume and age in 54 patients aged 0–17 years (median 2.0) with true-fast imaging with steady-state precession (FISP) sequences; in the presence of cysts, pineal parenchymal volume was defined as pineal gland volume minus cyst volume. The authors showed that the solid pineal parenchymal volume correlated more strongly with age than did the cystic pineal parenchymal volume [10]. The data of the study by Bumb et al. showed an increase of pineal volume with age, which was especially strong if only solid parenchyma is included (Pearson’s correlation coefficient *r* = 0.66); actually, the solid pineal parenchymal volume showed a mean of 3.9 mm^3^ (SD 2.8) at less than 1 year, 20.4 mm^3^ (SD 17.2) at 1–2 years, 21.0 mm^3^ (SD 16.7) at 2–4 years, and 40.0 mm^3^ (SD 24.1) at 5–11 years.

Bumb et al. suggested a possible bias in measuring pineal glands when including glands with a cystic component. Even though Bumb et al. [10] used a very reliable 3D evaluation method, in our study (based on T2-weighted sagittal 2D images with a slice thickness of ≤2 mm) we looked at a narrower age interval of the subject population, which only included the ages from 0 to 5 years. This, together with our far higher number of patients (184 versus about 30), allowed for a higher statistical significance of pineal gland size in that age range.

### Compared with pineal trilateral retinoblastoma

Normal gland sizes might also be used outside the context of retinoblastoma, but for retinoblastoma patients, knowledge of the normal gland could help with early detection of pineal trilateral retinoblastoma. In this situation, especially the solid (non-cystic) asymptomatic trilateral retinoblastomas—since patients with asymptomatic disease showed much better survival after pineoblastoma than did patients with symptoms [2]—are of interest as we restricted this study to the size of the normal solid pineal gland. Most asymptomatic solid pineoblastomas were above the upper bound normal pineal gland width, suggesting that the results of this study could indeed be useful to differentiate normal from abnormal solid pineal glands. We compared the maximum diameter (in any direction) of pineoblastoma with the upper 99 % prediction bound of the width of a normal gland, which is actually a conservative measurement in some cases, as some of the measurements of pineoblastoma actually might better be compared to the height of the lesion and should therefore be compared to the upper 99 % prediction bound of the gland height.

### Limitations

We have to address some limitations of our study. First of all, owing to the technical parameters chosen in our study, we only obtained the area of the glands, and true three-dimensional volumes of the glands could not be assessed. We assumed measurements from the transversal plane not to be statistically different with those from the sagittal plane, based on the measurements performed in a previous study in a series of 277 pineal glands [12]. Moreover, true-FISP sequences used by Bumb et al. [10] are not available in all MR units, so we used the universally available T2-weighted TSE sequence with a slice thickness 0.75–2 mm to measure the size of the pineal glands. This allowed for obtaining reliable measurements and the same certainty regarding pineal cysts (lower limit of diameter 2 mm) as was shown in the FISP sequences.

Second, small sample sizes may result in a potential large error in the estimates, causing wider prediction intervals than the estimated extent of individual variation, because it is affected by the uncertainty of sample estimates of mean and variance [20].

This study provides upper bounds for the sizes of normal pineal glands of patients aged 0–5 years, helping radiologists to decide whether they are within or outside the range of a clinical population. Of course, this does imply that a pineoblastoma will not be detectable based on pineal gland size until it has reached a certain size. Knowledge of the size of the normal pineal gland is helpful for detection of pineal gland abnormalities, particularly pineoblastoma.

## Electronic supplementary material

Below is the link to the electronic supplementary material.

ESM 1 (PDF 103 kb)
