# Supplementary material for: MRI-based assessment of the pineal gland in a large population of children aged 0–5 years and comparison with pineoblastoma: part I, the solid gland
Source: Neuroradiology. 2016 Apr 29;58:705–12. doi: 10.1007/s00234-016-1684-z (PMC4958126; doi:10.1007/s00234-016-1684-z)
Supplement: Supplementary file 1 — (PDF 103 kb) [file 234_2016_1684_MOESM1_ESM.pdf]

ONLINE APPENDICES

Appendix A. Two-way analysis of variance of gland sizes across age categories by gender

**Width§** ***(Lavene's test: p=0.21; adjusted R^2=0.19)***	**Sum of squares**	**Degrees of freedom**	**Mean square**	***F***	**P value**
Gender	0.10	1	0.10	1.03	0.31
Age	4.20	2	2.10	21.93	<0.0001
Gender*age	0.19	2	0.09	0.97	0.38
Within (error)	17.06	178	0.10		
Total	466.41	184			
**Height** ***(Lavene's test: p=0.57; adjusted R^2=0.14)***	**Sum of squares**	**Degrees of freedom**	**Mean square**	***F***	**P value**
Gender	0.54	1	0.54	0.70	0.41
Age	22.75	2	11.38	14.74	<0.0001
Gender*age	0.95	2	0.48	.62	0.54
Within (error)	137.43	178	0.77		
Total	1844.05	184			
**Area†** ***(Lavene's test: p=0.072; adjusted R^2=0.22)***	**Sum of squares**	**Degrees of freedom**	**Mean square**	***F***	**P value**
Gender	0.15	1	0.15	0.20	0.66
Age	40.84	2	20.42	26.20	<0.0001
Gender*age	1.93	2	0.97	1.24	0.29
Within (error)	138.70	178	0.78		
Total	2324.86	184			

§Weight was log transformed to meet the homoscedasticity assumption.
†Square root of area was taken to meet the homoscedasticity assumption.

Appendix B. Post hoc tests of the difference between age categories: Tukey's honestly significant difference test

Age categories (months)	Age categories (months)	Width (p value)	Height (p value)	Area (p value)
[0, 20)	[20, 40)	<0.0001	<0.0001	<0.0001
	[40, 60]	<0.0001	<0.0001	<0.0001
[20, 40)	[0, 20)	<0.0001	<0.0001	<0.0001
	[40, 60]	0.99	0.97	0.97
[40, 60]	[0, 20)	<0.0001	<0.0001	<0.0001
	[20, 40)	0.99	0.97	0.97
